# Commercially Available Phototherapy Devices for Treatment of Depression: Physical Characteristics of Emitted Light

**DOI:** 10.1176/appi.prcp.2019.20180011

**Published:** 2019-10-03

**Authors:** Mark A. Oldham, Mary B. Oldham, Paul H. Desan

**Affiliations:** ^1^ Department of Psychiatry University of Rochester Medical Center Rochester New York; ^2^ Sacred Heart Academy Hamden Connecticut; ^3^ Department of Psychiatry Yale School of Medicine New Haven Connecticut

**Keywords:** Phototherapy, Bright light therapy, Seasonal pattern, Seasonal affective disorder, Depressive disorders

## Abstract

**Objective:**

The purpose of this study was to evaluate key physical properties of commercially available light devices for the treatment of seasonal or nonseasonal depression and to determine whether the devices met clinical criteria, derived from evidence‐based clinical guidelines, for generating adequate light at a reasonable distance, over a reasonable field of illumination, and with an adequate degree of user acceptability.

**Methods:**

Twelve manufacturers loaned or donated 24 light therapy devices: 16 light boxes, one light column, four light‐emitting diode beam devices, and three light visors. Each device was evaluated for spectral power distribution, light dispersion, subjective discomfort from glare, adequacy of diffusion, photopic illuminance (in lumens per square meter [lux]), melanopic illuminance relative to photopic illuminance (efficacy ratio), and blue light hazard relative to melanopic illuminance (protection ratio).

**Results:**

Physical properties of emitted light varied widely among devices. Only seven larger light boxes satisfied the three clinical criteria. Some devices advertised as “10,000‐lux” devices produced this intensity only at unreasonably close distances, over a restricted field, or with unacceptable glare or unevenness of illumination. Five other devices emitted light with physical properties whose efficacy is less supported by research, although these devices may be useful for some patients.

**Conclusions:**

These results should help clinicians identify appropriate devices for patients seeking light therapy for seasonal or nonseasonal depression. Device selection is key to ensuring that patients receive evidence‐supported doses of light.

Bright‐light therapy is efficacious for major depressive disorder with and without a seasonal pattern, both as monotherapy and as an adjunct to antidepressant medication (1, 2, 3, 4). Since 2005, the American Psychiatric Association has recommended bright‐light therapy as a treatment option for patients with major depressive disorder (5). Research continues to expand its therapeutic application. A recent randomized trial found midday bright‐light therapy at 7,000 lumens per square meter (lux) to be efficacious as an adjunct to mood stabilizer for bipolar depression, with a 68% remission rate over 4–6 weeks, compared with a 22% remission rate with dim red placebo light (6). A randomized trial in antepartum depression found a 69% remission rate at 5 weeks with bright‐light therapy of 7,000 lux compared with a 36% remission rate with dim red placebo light (7). Despite its efficacy, the therapy remains underutilized, with “limited knowledge” among the most cited reasons for nonuse in a survey of psychiatrists (8). In this article, we evaluate commercially available bright‐light therapy devices to assess key physical properties of the light emitted and to inform clinicians about device selection.

Guidelines for bright‐light therapy (Box 1) typically recommend 10,000 lux for at least 30 minutes each morning (5, 9, 10), as summarized by Terman and Terman (11). Photopic lux is a unit of illuminance, the amount of perceived light per unit area in the light‐adapted human eye. The human retina is not equally sensitive to all wavelengths, with green and yellow photons appearing brighter than red or blue photons. Total illuminance of a light source containing photons of different wavelengths is calculated by multiplying energy at each wavelength by the retina's efficiency at converting energy to brightness and then by adding these products. For scale, a very dim overcast day might produce 100 lux to an observer outdoors, an overcast day 1,000 lux, a clear day 10,000–30,000 lux, and direct sun greater than 30,000 lux. A typical home interior might yield 50–200 lux, and an office 200–400 lux. Lux is a measure of light exposure at a particular location in relation to a source and is not an intrinsic property of a light source; a typical light source generates more lux for an observer closer to the source than for an observer farther away, and more lux for an observer centered in front of the source than for an observer who is off center.

**BOX 1. Ten guidelines for morning light box therapy**

- Because of the risk of activating suicidal or manic states, light therapy should be done only under the supervision of a qualified clinician.
- Use the device for 30 minutes every morning. Some patients will require longer treatment, and some patients can shorten treatment once a therapeutic response is obtained.
- Begin treatment at a consistent time each morning, as soon as possible after awakening. Complete treatment before 8:00 a.m.
- Patients who arise late will have to begin treatment at a later hour; with time, it should become easier to awaken earlier and complete treatment before 8:00 a.m.
- On weekends, do not delay treatment by sleeping in or omitting treatment.
- Use the device at the recommended distance to obtain 10,000 lux. A length of string can be used to help maintain the correct distance. If the recommended distance is not tolerable, try a slightly greater distance to reduce brightness.
- Do not look directly at the device but keep the device in the field of view. Using legs or another system to elevate the device and point it downward may increase convenience and minimize glare.
- If feeling overstimulated, reduce treatment length by 50% for a few days.
- Keeping a daily log of treatment start and stop times will help monitor and encourage adherence.
- We recommend a trial of 30 minutes every day for 4 weeks to start. Many patients do not adhere to treatment consistently and conclude prematurely that light therapy is not effective for them. After a strong response has been obtained, patients can try a shorter duration of treatment or skip some days of the week.

Many light therapy devices are available online to consumers and are advertised for their antidepressant effects. A device advertised as producing 10,000 lux may fail to specify the distance—which may be only inches from the device—at which it generates this intensity. Illustrations of smiling patients using light devices at much greater distances than recommended are common. Occasionally, the patient is pictured facing away from the device. Such images may create misconceptions for patients about proper use of light therapy, compromising its effectiveness. The U.S. Food and Drug Administration has not regulated the sale of bright‐light therapy devices. One pioneering U.K. study by Baczynska and Price emphasized the diversity of intensity, spectral characteristics, and directionality across 18 devices and the need to clarify the optimal parameters for phototherapy (12).

In this study, we evaluated key physical properties of the light emitted from bright‐light therapy devices that affect whether they are able to satisfy current evidence‐based clinical guidelines. We performed spectrophotometric evaluation of 24 commercially available devices and used these data to determine whether the device met three clinical criteria: did the device generate adequate photopic lux at a reasonable distance, over a reasonable area radially off center, and with an adequate degree of user acceptability? Additionally, because the circadian and antidepressant activity of light is surmised by some to be mediated by melanopsin‐containing, intrinsically photosensitive retinal ganglion cells, which are preferentially activated by short‐wavelength (blue‐appearing) light, we applied the melanopsin absorption spectrum in our calculation of melanopic lux (13). Because blue wavelengths pose greater retinal risk, we also calculated an estimate of the blue light hazard (14).

## Methods

Twenty‐four commercially available bright‐light therapy devices were donated or loaned by 12 manufacturers. For each device, we used a Jaz spectrophotometer from Ocean Optics (Largo, Florida), calibrated by the supplier before shipping, to measure illuminance in lux (10‐degree coefficients) and spectral power distribution (SPD) at the manufacturer‐recommended distance (MRD) with the probe centered over the light‐emitting surface (aperture) of the device. When the MRD was a range, we used the midpoint.

We evaluated light dispersion by measuring illuminance at 2‐inch intervals across a 24‐inch by 16‐inch grid parallel to the device's aperture at the MRD. We studied the effect of distance on illuminance by measuring illuminance at 6‐inch intervals from the aperture surface to 30 inches away (except for visor devices, which maintain a fixed distance when worn). We calculated the surface area of each device aperture and defined devices with an aperture ≥75 square inches as large and <75 square inches as small. Fourteen volunteers rated discomfort from glare on a Likert scale from 1 to 5, with higher scores indicating greater discomfort, for each device at the MRD. Study authors assessed whether an adequate diffuser was used to evenly illuminate the aperture of each device.

We tested whether each device met three clinical criteria. First, did the device produce adequate light at a reasonable distance? Adequate light was defined as ≥7,000 lux at the MRD (or at 12 inches, if the MRD was less than 12 inches), because recent research has successfully used 7,000‐lux interventions (6, 7). Second, was the field of illumination reasonable for the user? We defined “reasonable” as allowing a user to move 6 inches radially off center within the tangent plane at the MRD and still receive ≥5,000 lux; we considered a 1‐foot diameter circle a minimally sufficient area for use. We chose 5,000 lux as the threshold to ensure a dose of light that has been used in previous studies of bright‐light therapy (11). Third, was the experience acceptable for the user? We defined “acceptability” as a glare rating ≤3.75, which was the consensus value achieved through discussion with the volunteers trying out the devices for our study. Values above this rating were considered unpleasant by the volunteers, even for brief exposures of several minutes. We also required an adequate diffusion screen with no obvious intense areas (hot spots) that risked exposing focal areas of the retina to increased light energy.

We converted photopic illuminance to melanopic lux, an estimate of melanopsin activation based on the opsin absorption nomogram, with a peak absorption near 480 nm, or 484 nm after correction for transparency of human optical media. We imported raw SPD values (microwatts/square cm/nm) into a published Excel calculator (13). We reported the efficacy ratio suggested by Baczynska and Price (12), the ratio of melanopic lux to photopic lux—that is, the ratio between postulated therapeutic efficacy and apparent brightness. Finally, because light in the blue wavelengths is more toxic to the retina than light in other wavelengths, we used a model based on primate research to estimate the blue light hazard. Observed irradiance over 5‐nm steps was multiplied by the blue light hazard function, and the products were summed to estimate the blue light hazard (14). We calculated the protection ratio suggested by Baczynska and Price (12), the ratio of melanopic lux to blue light hazard normalized by dividing by 10—the ratio between postulated therapeutic efficacy and estimated retinal risk.

## Results

**Figure 1:**
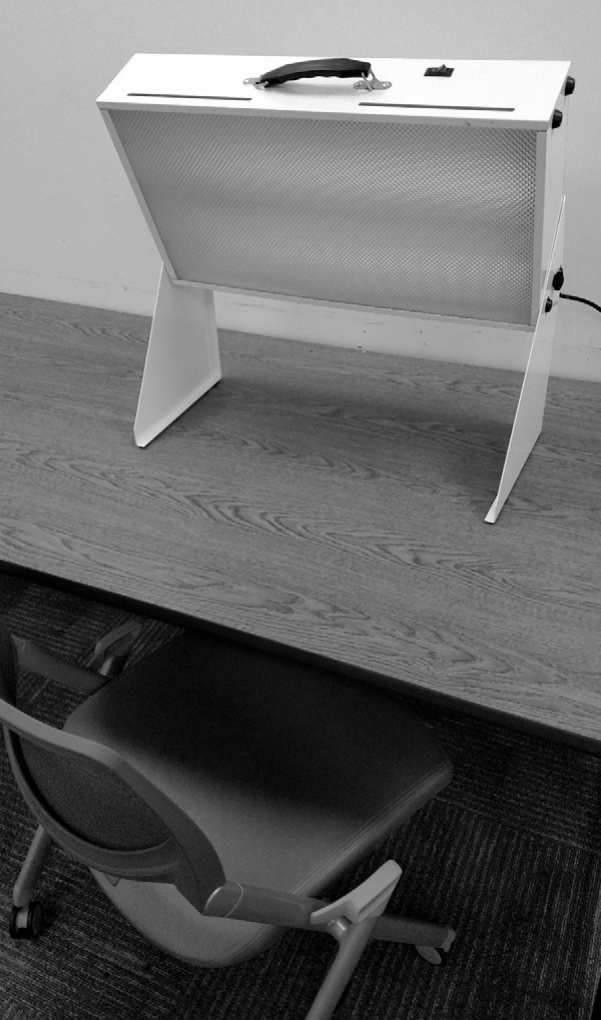
Typical 10,000‐lux light box as used in most research (device X3)^a^

We evaluated 24 devices (Table 1): 16 light boxes (X1–X16), one column device (C1), four light‐emitting diode (LED) beam devices (M1–M4), and three head‐mounted visors (V1–V3). Eleven light boxes (X1–X11) were large (i.e., aperture ≥75 square in), and six of these had built‐in or optional legs to elevate the box to eye level or above (X1–X6). Elevated devices reduce glare, preferentially stimulate the ventral retina, and free up space for the user to eat breakfast, read, or conduct other activities at a table or counter. A typical large light box with legs (X3) is illustrated in Figure 1. One of the large box devices (X7) and two of the small box devices (X13 and X14) were desk lamps whose light fixture could be tilted up for use as an eye‐level light box or tilted down for use as a desk lamp. The remaining boxes were intended to be placed on a table or other flat surface. Light boxes are listed in Table 1 by type and in order of decreasing aperture area. The column device is notable for its small tabletop footprint. The four LED beam devices we tested were more compact than traditional light boxes and produced narrower beams of light and smaller fields of illumination. The three visor devices produced a fixed region of illumination on the eyes regardless of patient movement.

a

**Table 1:** Physical properties of 24 light therapy devices for treatment of seasonal or nonseasonal depression^a^

								**Glare rating** ^i^					
**Device**	**Type** ^b^	**Light source** ^c^	**Area (in^2^)** ^d^	**MRD (in)** ^e^	**Photopic illuminance (lux)** ^f^	**Efficacy ratio** ^g^	**Protection ratio** ^h^	**M**	**SD**	**≥7,000 lux at center** ^j^	**≥5,000 lux off center** ^k^	**Glare rating(< 3.75)** ^l^	**Adequate diffusion** ^m^
X1	Box, large, elevated	Fluorescent, cool	275	23	11,800	.76	1.00	3.61	.30	+	+	+	+
X2	Box, large, elevated	Fluorescent, cool	275	18	8,930	.68	1.21	2.75	.24	+	+	+	+
X3	Box, large, elevated	Fluorescent, warm	242	24	9,010	.59	1.09	3.54	.27	+	+	+	+
X4	Box, large, elevated	Fluorescent, warm	208	12	10,900	.57	1.30	3.00	.21	+	+	+	+
X5	Box, large, elevated	Fluorescent, warm	174	16	7,150	.48	1.17	2.36	.20	+	+	+	+
X6	Box, large, elevated	Fluorescent, warm	134	12	11,200	n/d	n/d	2.86	.23	+	+	+	+
X7	Desk lamp, large	Fluorescent, warm	134	18	6,580	.51	1.16	3.21	.30	+	+	+	–
X8	Box, large	Fluorescent, cool	115	30	8,010	.94	1.25	3.50	.34	+	+	+	–
X9	Box, large	Fluorescent, warm	90	12	8,150	.45	1.25	2.85	.15	+	–	+	+
X10	Box, large	LED, white	79	24	11,100	.88	.98	4.32	.30	+	+	–	–
X11	Box, large	Fluorescent, cool	76	14	9,820	1.28	.90	3.08	.23	+	+	+	+
X12	Box, small	Fluorescent, cool	72	14	11,900	.75	1.04	3.89	.32	+	+	–	+
X13	Desk lamp, small	Fluorescent, cool	63	18	7,780	.95	1.14	3.07	.36	+	+	+	–
X14	Desk lamp, small	Fluorescent, cool	60	14	11,600	.74	1.01	3.89	.24	+	+	–	–
X15	Box, small	Fluorescent, cool	38	14	11,200	.76	1.03	4.50	.23	+	+	–	–
X16	Box, small	Fluorescent, cool	36	12	4,600	.84	1.09	2.86	.23	–	–	+	+
C1	Column	Fluorescent, green	80	24	510	2.35	6.35	n/d		–	–	+	+
M1	Beam	LED, white	10	20	3,750	1.24	1.18	3.25	.41	–	–	+	+
M2	Beam	LED, white	35	24	1,110	1.02	.95	3.07	.36	–	–	+	–
M3	Beam	LED, white	14	20	2,020	.86	.83	3.96	.31	–	–	–	–
M4	Beam	LED, blue	35	24	51	34.5	.13	2.04	.35	–	–	+	–
V1	Visor	LED, green	n/a	n/a	8,900	2.37	5.39	n/d		+	n/a	n/a	–
V2	Visor	LED, white	n/a	n/a	13,800	1.33	1.41	n/d		+	n/a	n/a	+
V3	Visor	LED, white	n/a	n/a	5,900	1.33	1.20	n/d		–	n/a	n/a	–

a n/a, not applicable; n/d, not done; +, satisfies criterion; –, does not satisfy criterion.

b Devices were classified as “large” if their area was ≥75 in^2^ and “elevated” if legs are provided to raise the device.

c Light source indicates “warm” or “cool” fluorescent, white light‐emitting diode (LED), or colored LED.

d Area is the approximate size of the aperture in square inches.

e MRD is the manufacturer‐recommended distance for use or 12 inches, whichever is greater.

f Photopic illuminance is measured at the MRD.

g Efficacy ratio is the ratio between melanopic lux and photopic lux at the MRD.

h Protection ratio is melanopic lux divided by estimated blue light hazard.

i Glare ratings are the mean±SD of ratings by volunteer raters.

j ≥7,000 lux at center indicates that the device generated greater than or equal to 7,000 lux at the MRD. (Device X7 was considered to do so because it met this threshold at a slightly smaller distance.)

k ≥5,000 lux off center indicates that the device generated greater than or equal to 5,000 lux 6 inches off the central axis at the MRD.

l Glare rating (<3.75) indicates a mean glare rating <3.75 on a Likert scale from 1 to 5, with higher scores indicating greater user discomfort from glare. (Device C1 was not tested but is not glaring and was judged likely to meet this standard.)

m Adequate diffusion means no significant hotspots of light intensity were noticeable.

### Spectrophotometric Analysis

**Figure 2:**
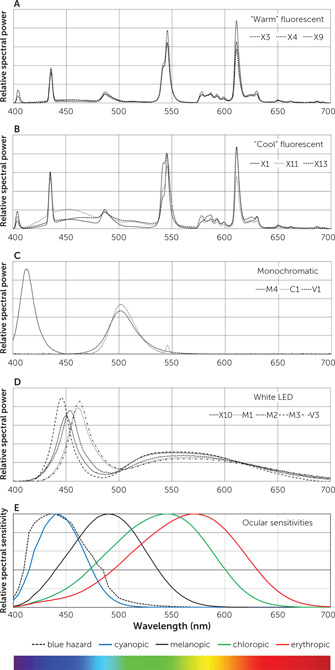
Spectral power distribution (SPD) curves for sample bright‐light therapy devices and human ocular sensitivity curves^a^

We identified four SPD patterns (Figure 2). Fluorescent devices emitted one of two spectral signatures: a “warm” color‐temperature with peaks of emitted energy (e.g., X3, X4, X9) (Figure 2a) and a “cool” color‐temperature with similar peaks but increased energy in short and middle wavelengths (e.g., X1, X11, X13) (Figure 2b). Monochromatic devices included one blue‐light LED device peaking around 413 nm (M4) and two green‐light devices, one with an LED (peaking at 501 nm; V1) and one with a filtered fluorescent source (peaking at 503 nm; C1) (Figure 2c). White LED devices (X10, M1, M2, M3, V3; V2 was similar to V3) emitted a short‐wavelength peak in the short (blue‐appearing) wavelengths with a lower, broader peak in the middle wavelengths, generating light that appeared bluish‐white (Figure 2d). Among these devices, there was a range of wavelengths at peak emission: 450 nm for X10, 461 nm for M1, 453 nm for M2, 445 nm for M3, and 463 nm for V3. The SPD patterns are shown relative to the sensitivities of the three cone‐specific photoreceptors, the melanopic absorption curve, and the blue hazard curve (Figure 2e).

a

### Adequacy of Light at MRD

**Figure 3:**
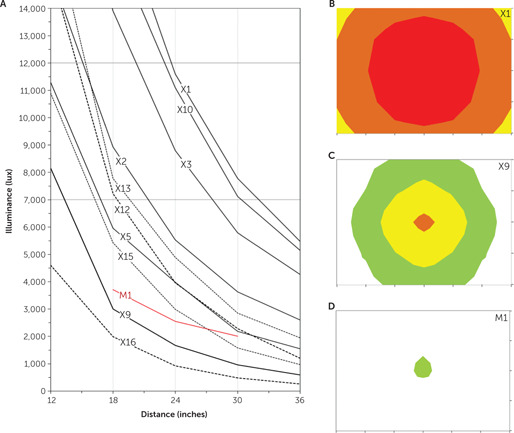
Relationship between illuminance and distance and between illuminance and position for sample bright‐light therapy devices^a^

Table 1 lists illuminance at the MRD for each device. Most light boxes produced ≥7,000 lux at the MRD. Light intensity received by the observer decreased with increasing distance from the device (Figure 3a). The larger devices were brighter and produced ≥7,000 lux over a wide range of distances. For example, device X3 produced 12,000 lux at a distance of about 20 inches and 7,000 lux at a distance of approximately 28 inches. Smaller devices produced less intense light. For example, device X9 produced 7,000 lux at a distance of 13.5 inches, but a user any farther from this device would receive less illuminance than our minimum. Of the LED devices, one produced 3,750 lux at the MRD, but the other three produced substantially dimmer illuminance (Table 1). The three visor devices,V1, V2, and V3, produced 8,900, 13,800, and 5,900 lux, respectively, when assessed at 1.5 inches—the approximate exposure distance when worn. Our measurements generally agreed with manufacturer specifications where provided.

a

### Field of Light Radially Off Center

All but one of the large light boxes and all but one of the small boxes emitted ≥5,000 lux 6 inches radially off center in all directions (Table 1). Figure 3b shows the intensity distribution in the plane of the observer for a light box that met this criterion (X1); Figure 3c shows one that did not meet this criterion (X9); Figure 3d shows the narrow field produced by a beam device (M1); Figure S1 in the online supplement is a three‐dimensional illustration of how illuminance decreased with movement off center of the plane of the observer for one large box (X4).

### Experience Acceptability

Mean±SD glare ratings by the volunteers are shown in Table 1. There was limited correlation between glare and illuminance; at a given illuminance, some devices produced much more glare than others. As a group, small light boxes were most likely to be perceived as glaring (i.e., score≥3.75), with three of these five devices considered uncomfortable to look at. We considered many devices to have inadequate diffusion with noticeable hot spots. None of the devices evidenced subjective flicker.

### Melanopic Lux

If melanopic lux represents an estimate of the therapeutic effect of a particular SPD, the efficacy ratio, or melanopic lux divided by photopic lux, represents the postulated therapeutic effect per unit of perceived brightness for a given source. Table 1 shows that the efficacy ratios of warm fluorescent devices were generally lower than those of cool fluorescent devices (mean=0.52, range 0.45–0.59, versus 0.86, 0.68–1.28, respectively). White LED devices preferentially emitted light with shorter wavelengths, resulting in higher efficacy ratios (mean=1.11, range 0.86–1.33). The two green‐emitting devices had high efficacy ratios of 2.35 and 2.37, as did the one blue‐emitting LED device (34.5). In this melanopic model, cool fluorescent sources appeared to have higher efficacy ratios than warm fluorescent devices, and the green and blue monochromatic sources had the highest efficacy ratios.

### Blue Light Hazard

In Table 1, we report a protection ratio for each device, defined as the ratio of melanopic lux to blue light hazard, representing postulated therapeutic benefit per unit of hazard. Warm fluorescent devices had slightly higher protection ratios (mean=1.19, range 1.09–1.30) than cool fluorescent devices (mean=1.07, range 0.90–1.25). White LED devices (mean=1.09, range 0.83–1.41) showed a broader range, reflecting differing patterns of short‐ versus middle‐wavelength energy, as illustrated in Figure 2d. The two green‐emitting devices had high protection ratios of 5.39 and 6.35, as these devices emit very little short‐wavelength light. The deep‐blue‐emitting LED device had the worst protection ratio, 0.13, which could be expected from its peak energy emission at 413 nm.

## Discussion and Conclusions

Multiple studies have supported the efficacy of morning bright‐light therapy from large light boxes in the treatment of patients with seasonal or nonseasonal depression. These devices generate 10,000 lux of broad‐spectrum white light at a reasonable distance over a large area. In our survey of 24 devices, we identified only seven that met our criteria of ≥7,000 lux at ≥12 inches and ≥5,000 lux at 6 inches radially off center, without glare or hot spots: four large light boxes (similar to those used in previous research) that easily exceeded these criteria and three smaller light boxes that minimally met these criteria. We also identified an additional five devices with physical properties whose efficacy is supported by limited research: one LED beam unit, three visor units, and one column unit. Our results should help clinicians discuss device options with their patients.

### Light Boxes

Most evidence supporting the efficacy of bright‐light therapy is based on large 10,000‐lux white (broad‐spectrum) light boxes, such as X1–X4. Clinicians can inform their patients that these four devices are comparable to devices used in research and meet clinical guidelines for light therapy. X1–X3 produce 10,000 lux over a large area. X4 produces 10,000 lux at a shorter distance but has legs that permit easy use at this shorter distance and has been validated by research (6, 7). Clinicians may also indicate that light boxes such as X5, X6, and X11, are smaller options that may offer increased convenience but constrain user experience. There are free lux‐measurement cell phone apps that permit patients to verify they are receiving 10,000 lux at a specific distance from the light source.

### LED Beam Units

These devices emit light with energy relatively concentrated in the shorter (blue‐appearing) and middle (green‐appearing) wavelengths, to which the human circadian system is particularly sensitive (15). The retinal pigment melanopsin maximally absorbs such wavelengths and activates intrinsically photosensitive retinal ganglion cells, which project directly to the suprachiasmatic nucleus, the master clock of the circadian rhythm system (16). Recent research in mice suggests that the mood‐enhancing effects of light may be due to a subset of such ganglion cells projecting to the perihabenular region (17). One small trial demonstrated therapeutic efficacy in seasonal affective disorder for bright green light compared with dim red light (18). Placebo‐controlled trials of LED devices for patients with the disorder have shown efficacy for 398‐lux narrow‐band blue light (19), for 200‐lux narrow‐band blue light (20), and for 1,350‐lux blue‐enriched white light (21). A trial comparing 98‐lux narrow‐band blue light with 711‐lux blue‐enriched white light found similar therapeutic effect in patients with seasonal affective disorder (22), and a trial of 100‐lux narrow‐band blue light versus 10,000‐lux bright white light found similar effect in patients with subsyndromal seasonal affective disorder (23). Given the absence of a placebo condition in the latter two studies, these findings should be considered preliminary. In general, however, the response rates found in these studies were similar to those of trials with 10,000‐lux white light boxes. Together, these studies suggest that short and middle wavelengths are particularly effective for patients with seasonal affective disorder.

Of the devices we tested, M1 was found to be efficacious for the treatment of seasonal affective disorder in a randomized trial with 106 participants (24). The other three beam devices we tested provided much less intensity of exposure and were judged to be glaring or inadequately diffused. Device M4 emitted very short‐wavelength light (413 nm peak), which not only increases blue hazard but is lower than the peak absorption wavelength of the melanopsin system. This device had the worst protection ratio of devices studied. Light in the deep‐blue regions of the spectrum may offer a poor balance of therapeutic potential and retinal risk.

Certain patients nevertheless prefer beam devices (including M1 and other LED devices not included in this study) because of their portability and compact design, provided there is adequate diffusion to reduce glare. Clinicians may discuss a trial of such devices to offer certain practical advantages to patients, but patients need to know they must position these devices carefully to keep the light beam focused on the eyes, typically around 30 degrees from the line of sight. None of these devices produce 10,000 lux at MRD, and it is unknown whether different intensity requirements for efficacy apply to these small concentrated light sources than to large light box sources.

### Visor Devices

Early attempts at using light visors as therapy for seasonal affective disorder were unsuccessful, but the visors in published controlled trials emitted white incandescent light with most energy at long wavelengths (25). A promising open‐label 4‐week trial of an LED visor similar to V3 found full recovery (score <8 on the Structured Interview Guide for the Hamilton Depression Rating Scale–Seasonal Affective Disorder Version, a Hamilton Depression Rating Scale modified to reflect atypical neurovegetative symptoms of seasonal affective disorder) in 10 of 11 patients at the trial's end. Although open‐label, these preliminary data suggest that a visor device may provide adequate light to serve as a therapeutic option (26). We are unaware of any published placebo‐controlled trial of an LED visor device. Of the three visor devices we studied (V1–V3), two provided ≥7,000 lux, and the third provided almost 6,000 lux. V1 emitted middle‐wavelength, green‐appearing light; V2 and V3 emitted bluish‐tinted white light. All three visor devices were relatively glaring and warrant better diffusion. Because of their convenience, visor devices may interest certain patients. In discussing such devices with patients, the clinician should instruct the patient to adjust the visor device with a mirror to ensure direct illumination of the eyes, because patients are likely to reduce glare by misadjusting the device.

### Column Devices

The one column device (C1) we studied—one of only two green‐light devices in the study—produced minimal glare and had a small tabletop footprint. Prior research found that a similar device phase shifted circadian rhythms similarly to a 10,000‐lux light box (27) and potentiated the antidepressant effect (28). These previous studies, however, used two columns, so patients would need to purchase two columns or a double column unit to replicate those effects. In discussing this option with patients, clinicians should indicate that further research is needed with this type of device.

### Study Limitations

We tested only a portion of the commercially available devices, and failure to include a device should not be taken as a negative recommendation. Some manufacturers declined to provide devices for testing, and others submitted only part of their product line. Additionally, given the apparent trade‐off in intensity and duration (11), dimmer devices may be effective if used for longer periods, but the required treatment duration may be prohibitively inconvenient. Conversely, satisfying our three clinical criteria may suggest but does not guarantee efficacy for a device: such a guarantee would require an adequate clinical trial.

An unavoidable limitation to our study was that inadequate research has been done regarding optimal wavelengths for bright‐light therapy. Most devices in this study emitted broadband white or bluish‐white light with protection ratios near 1. One device emitted very deep‐blue light resulting in a much lower protection ratio because of increased blue light hazard. There is no evidence that treatment efficacy is greater with short‐wavelength light than with adequate levels of broad‐spectrum white light, and devices that emit high levels of short‐wavelength light may pose unnecessary risk to the retina. Two devices in the study emitted green light with notably higher protection ratios. Figure 2e illustrates the comparative sensitivities of the cyanopic (blue), chloropic (green), and erythropic (red) cone systems, the melanopic system, and blue light hazard estimated from primate data. If melanopic lux predicts antidepressant response, then green‐appearing light in the 500–540 nm range may provide the best balance of therapeutic efficacy and safety. Research on the therapeutic efficacy of light in the green wavelengths is urgently needed. Our results echo the conclusions of Baczynska and Price (12): marketed light therapy devices represent a wide range of intensities, directionalities, and spectral properties, and only limited research is available to clarify optimal parameters for light treatment.

Clinicians should note that the U.S. Food and Drug Administration has yet to approve any light therapy device for depression and has not regulated such devices. Despite evidence on its antidepressant efficacy, bright‐light therapy has not been studied to the same extent as pharmacological approaches. A properly diffused, conventional large light box exposes the eye to less light than being outdoors on a sunny day, and research suggests such boxes are ophthalmologically safe (29). However, there are limited data about the long‐term effects of bright‐light therapy in general, and no data regarding the safety of newer device types (30). Caution is needed in treating individuals with retinal disease, medical illness such as diabetes associated with retinal disease, and concurrent use of photosensitizing medications (11).

### Implications for Clinicians

Of the 24 devices tested, only four were similar (or identical) to the large light boxes used in most research on bright‐light therapy (X1–X4). Three devices were smaller light boxes that met our three criteria (X5, X6, and X11). Five devices were found to have properties supported by at least some research (M1, V1–V3, and C1). These devices are identified in Table S1 of the online supplement. Descriptions and photographs of these devices are provided on our institutional website (https://medicine.yale.edu/psychiatry/research/programs/clinical_people/winter.aspx).

Traditional light boxes, such as X1–X4, are generally preferred as a first‐line approach to bright‐light therapy, given that they are best supported by research. After response to the therapy is established, however, a trial of a smaller device that offers certain advantages, such as convenience, may be attractive to some patients. Clinicians should inform their patients that many devices marketed on the Internet do not produce 10,000 lux at a reasonable distance, over a reasonable area, with a reasonable degree of comfort. Clinicians should only recommend light boxes that specify the distance at which they produce 10,000 lux.

Our results should enable clinicians to understand what types of light devices are best supported by current research and to discuss options knowledgeably with patients. We urge clinicians to familiarize themselves with clinical guidelines (Box 1) for bright‐light therapy and to increase their use of this underutilized treatment for depression.

## Supporting information

Supplementary Material

## Footnotes

a This device generates 10,000 lux at 23 inches. The device is shown with optional legs, which raise the device, making it easier for the user to remain at the optimal distance from the device and allowing for table space in front of the user.

a Panel A illustrates typical SPD curves, indicating relative power at specific wavelengths, for devices with “warm” white fluorescent light sources. Panel B illustrates typical SPD curves for devices with “cool” white fluorescent light sources. These devices have increased energy in short and middle wavelengths, generating a more blue‐appearing light. Panel C illustrates SPD curves for devices with monochromatic light sources, either blue or green. Panel D shows SPD curves for devices with white light‐emitting diode (LED) light sources. These devices use LEDs with peak emission in blue wavelengths and a broader peak of emission in longer wavelengths. Light emitted by these devices appears bluish‐white. Panel E shows relative sensitivities of the human eye at specific wavelengths: the curves have been normalized to show equal peak sensitivity. Sensitivities of the cyanopic (blue) cones (blue line), melanopic system (black line), chloropic (green) cones (green line), and erythropic (red) cones (red line) are compared to estimated blue light hazard (black dashed line). If therapeutic efficacy is related to the melanopic curve, then light in the region of 500–540 nm may offer the best balance of efficacy and safety; as shown in the spectral depiction, this balance is the green region.

a Panel A illustrates relationships between illuminance (lux) and distance (inches) for large and small light box devices. Illuminance versus distance is also illustrated with a red line for a beam device (M1), showing a flatter decline in brightness with increasing distance. Panel B shows illuminance versus position in the plane of the observer at the manufacturer‐recommended distance (MRD) for a large light box (X1), which provides adequate intensity over a large region. Panel C shows illuminance versus position in the plane of the observer at the MRD for a smaller box (X9), which does not meet the criterion of providing ≥5,000 lux over a region of 6 inches radially off center. Panel D illustrates illuminance versus position in the plane of the observer at the MRD for a beam device (M1). The device requires careful alignment to ensure adequate light exposure. White, <2,500 lux; green, 2,500–5,000 lux; yellow, 5,000–7,500 lux; orange, 7,500–10,000 lux; Red, >10,000 lux. Area shown is 24 inches wide and 16 inches high.
