# Supplementary material for: Commercially Available Phototherapy Devices for Treatment of Depression: Physical Characteristics of Emitted Light
Source: Psychiatr Res Clin Pract. 2019 Oct 3;1(2):49–57. doi: 10.1176/appi.prcp.2019.20180011 (PMC9175704; doi:10.1176/appi.prcp.2019.20180011)
Supplement: Supplementary file 1 — Supplementary Material [file RCP2-1-49-s001.pdf]

Supplementary Figure SF1.

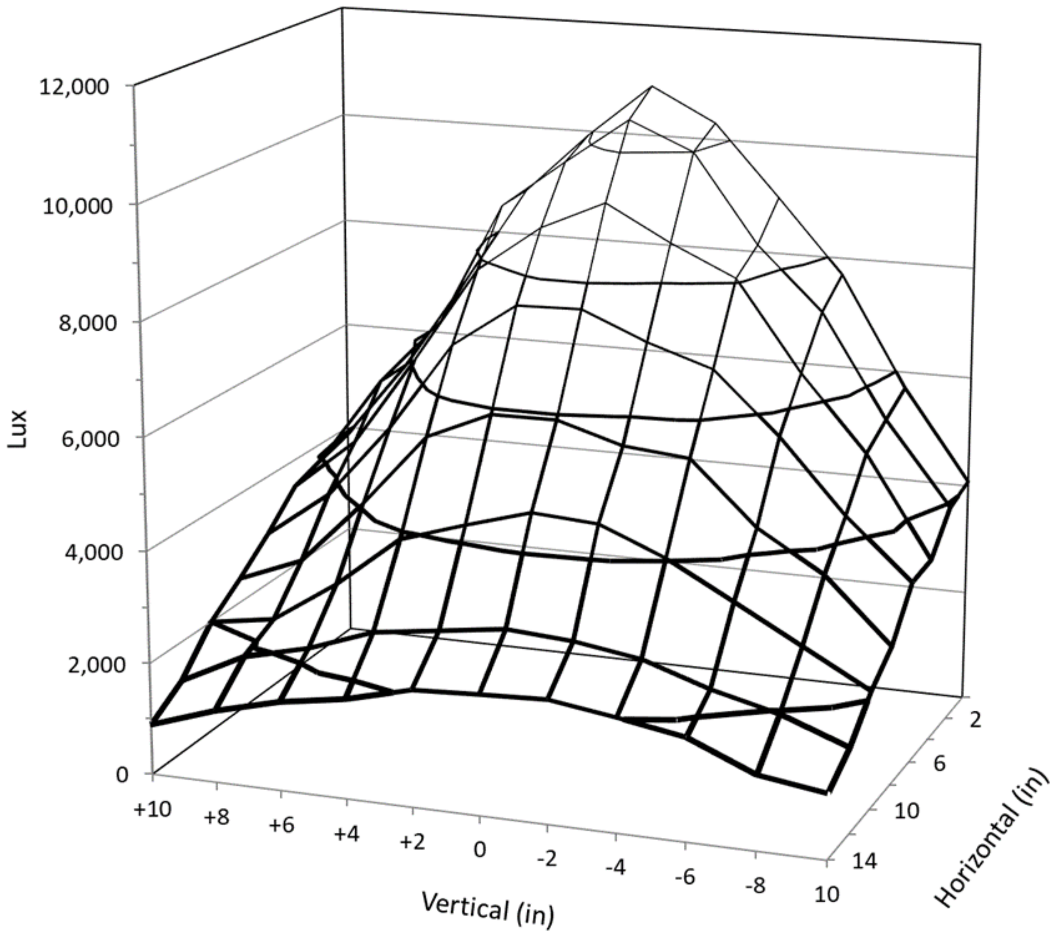

Supplemental Figure SF1. Illuminance (lux) versus position in the plane of the observer at MRD for device X4. Intensity declines with vertical displacement above or below the device axis or lateral displacement sideways from the device axis.

Supplemental Table ST1. Research-supported devices for SAD treatment. Model and manufacturer are shown for selected devices in this study.

FULL-SIZE 10,000 LUX LIGHT BOXES. These devices are similar to those used in most research studies on bright light therapy for Seasonal Affective Disorder (SAD). Device X2 does not appear to be marketed at this time.

Device X1. SunRay II
The SunBox Company, 201 Broadway Street, Suite 220, Frederick, MD 21701
800-548-3968, 240-651-3286
orders@sunbox.com
www.sunbox.com

Device X3. NorthStar 10,000
Alaska Northern Lights, Inc., 59 Damonte Ranch Pkwy, #B-262, Reno, NV 89521
800-880-6953, 775-852-8192
info@alaskanorthernlights.com
www.alaskanorthernlights.com

Device X4. Day-Light Classic (DL930)
Carex Health Brands, 921 E Amidon St., Sioux Falls, SD 57104
800-526-8051
info@carex.com
day-lights.com

SMALLER 10,000 LUX LIGHT BOXES. In our tests several smaller light boxes appeared to meet our specifications for 10,000 lux therapy. The user will need to sit closer and may not be able to move as much side-to-side. Some users may feel that the decrease in convenience would be offset by the more compact size of the device.

Device X5. BOXelite
Northern Light Technologies, Inc., 8971 Henri-Bourassa Blvd. West, Montreal, Qc H4S 1P7 Canada
800-263-0066, 514-335-1763
info@NorthernLightTechnologies.com
northernlighttechnologies.com

Device X6. Day-Light Sky (DL2000)
Carex Health Brands, 921 E Amidon St., Sioux Falls, SD 57104
800-526-8051
info@carex.com
day-lights.com

Device X11. SunTouch Plus
Nature Bright Company, 5251 California Ave Suite 130 Irvine, CA 92617
800-622-0231, 949-625-4900,
www.naturebright.com

LIGHT-EMITTING DIODE (LED) BEAM DEVICES. A smaller amount of research supports the use of small LED devices for SAD. Such devices are much more compact than conventional light boxes, may be battery powered, but must be carefully aligned to keep the eyes in the beam. The intensity of 10,000 lux does not seem to be required.

Device M1. Litebook Advantage
The Litebook Company LTD, #6, 941 South Railway St SE, Medicine Hat, Alberta T1A 2W3 Canada
877-723-5483, 403-504-1533
info@litebook.com
litebook.com

LIGHT VISORS. Four controlled clinical trials in the 1990s found no evidence that incandescent light visors are effective for SAD. Only one small uncontrolled trial of newer visors using LED sources has been published in abstract form, suggesting therapeutic effect in SAD. Device V1 is one of the two green light devices in the study.

Device V1. Feel Bright Light
Physician Engineered Products, 103 Smith Street, Fryeburg, ME 04037
800-622-6240, 207-935-1256
info@feelbrightlight.com
www.feelbrightlight.com

Device V3. SolarMax Light Visor
BioBrite, Inc, 4350 East-West Highway, Suite 520, Bethesda, MD 20814
301-961-5940
info@biobrite.com
www.biobrite.com/

LIGHT COLUMNS. An array of two columns was found to shift circadian rhythms similar to a 10,000 lux light box, and a similar double array has been used in other depression research. We are unaware of any controlled trial of the therapeutic effect of this device in SAD. Device C1 is one of the two green light devices in the study: one would need to purchase two columns or a double column device to duplicate the arrays used in circadian and depression research.

Device C1. Lo-LIGHT Therapy Lamp
Sunnex Biotechnologies, Suite 657, 167 Lombard Ave. Winnipeg, MB R3B0V3 Canada
877-778-6639, 204-956-2476
www.sunnexbiotech.com

Product names listed above may be registered trademarks.
